# Multimodal multiphoton imaging for label-free monitoring of early gastric cancer

**DOI:** 10.1186/s12885-019-5497-4

**Published:** 2019-04-02

**Authors:** Lianhuang Li, Deyong Kang, Zicheng Huang, Zhenlin Zhan, Changyin Feng, Yongjian Zhou, Haohua Tu, Shuangmu Zhuo, Jianxin Chen

**Affiliations:** 1 Key Laboratory of OptoElectronic Science and Technology for Medicine of Ministry of Education, Fujian Provincial Key Laboratory for Photonics Technology, Fujian Normal University, Fuzhou, 350007 People’s Republic of China; 2 Department of Pathology, Fujian Medical University Union Hospital, Fuzhou, 350001 People’s Republic of China; 3 Department of Gastroenterology, The First Hospital of Quanzhou Affiliated to Fujian Medical University, Quanzhou, 362002 People’s Republic of China; 4 Department of Gastric Surgery, Fujian Medical University Union Hospital, Fuzhou, 350001 People’s Republic of China; 5 Beckman Institute for Advanced Science and Technology, University of Illinois at Urbana-Champaign, Urbana, IL 61801 USA

**Keywords:** Early gastric cancer, Multiphoton imaging, Two-photon excited fluorescence, Second-harmonic generation

## Abstract

**Background:**

Early gastric cancer is associated with a much better prognosis than advanced disease, and strategies to improve prognosis is strictly dependent on earlier detection and accurate diagnosis. Therefore, a label-free, non-invasive imaging technique that allows the precise identification of morphologic changes in early gastric cancer would be of considerable clinical interest.

**Methods:**

In this study, multiphoton microscopy (MPM) using two-photon excited fluorescence combined with second-harmonic generation was used for the identification of early gastric cancer.

**Results:**

This microscope was able to directly reveal improved cellular detail and stromal changes during the development of early gastric cancer. Furthermore, two features were quantified from MPM images to assess the cell change in size and stromal collagen change as gastric lesion developed from normal to early cancer.

**Conclusions:**

These results clearly show that multiphoton microscopy can be used to examine early gastric cancer at the cellular level without the need for exogenous contrast agents. This study would be helpful for early diagnosis and treatment of gastric cancer, and may provide the groundwork for further exploration into the application of multiphoton microscopy in clinical practice.

## Background

Early gastric cancer is defined as gastric cancer that is confined to the mucosa or submucosa, regardless of the presence or absence of regional lymph node metastasis [1, 2]. The prognosis for early gastric cancer is universally excellent, and strategies to improve prognosis is strictly dependent on earlier detection and accurate diagnosis since early gastric cancer can potentially be cured by endoscopic therapy such as endoscopic mucosal resection or endoscopic submucosal dissection [3, 4]. It was reported that patients with early gastric cancer after treatment had a 1 year survival in excess of 90% [5]. However, there is little information on the symptoms of early gastric cancer and it is often difficult to detect gastric superficial lesions using conventional endoscopy with white-light imaging [6]. At present, the diagnosis of these lesions is always based on the pathologic assessment of endoscopic biopsy specimens. As such, superficially taken biopsies and sampling error for histopathology are common events, making it difficult to draw firm conclusions [7]. Hence, development of a new diagnostic technique will be of important clinical meaning.

Multiphoton imaging technique such as two-photon excitation fluorescence (TPEF) and second-harmonic generation (SHG) has been widely used for biological tissue imaging [8, 9]. TPEF exploits the auto-fluorescence of biological samples and therefore obviates the need for exogenous contrast agents, and SHG makes use of the non-centrosymmetric properties to image structural proteins and will not suffer from phototoxicity effects or photobleaching because it does not involve excitation of molecules [10–12]. There are a variety of intracellular molecules including NADH, FAD and porphyrins, as well as certain extracellular components such as elastin and collagen within gastric tissues, which can generate intrinsic multiphoton signals. Therefore, in this study, we investigated the potential of using label-free, multimodal multiphoton microscopy that incorporates two-photon excited fluorescence and second-harmonic generation techniques for distinguishing early gastric cancer from normal tissues.

## Methods

### Imaging instrumentation

As has been described previously, a mode-locked femtosecond Ti:sapphire laser (Mira 900-F, Coherent, Inc., USA) was used as the multiphoton excitation source, and the excitation light was delivered to and the emitted light was also collected from the sample through an inverted microscope (LSM 510 META, Zeiss, Germany) [13, 14]. A 63× Zeiss Plan-Apochromat oil immersion objective lens (Numerical Aperture (NA) =1.4) was chosen for capturing multiphoton microscopic images. The backscattered intrinsic TPEF and SHG signals were received respectively by a META detector composed of a reflective grating and an optimized 32-channel PMT array detector using 810 nm excitation wavelength. TPEF signal was detected in the wavelength range 430–716 nm and SHG signal was detected in the 389 to 419 nm wavelength range. In order to provide a visual contrast of multiphoton images, all the TPEF images adopted pseudo-colored red and SHG images were marked with pseudo-colored green.

### Sample preparation

In this work, 12 early gastric cancer samples including 1 well-differentiated adenocarcinoma, 6 moderately-differentiated adenocarcinomas, 1 moderately-poorly differentiated adenocarcinoma and 4 poorly-differentiated adenocarcinomas were collected. All samples were immediately sent to the pathology laboratory for frozen section through a cryostat microtome once they were obtained from the surgeons. Three consecutive sections with 10 μm thickness were used in this study, in which two slices were used to carry out the multiphoton microscopic imaging and the middle section was diagnosed through hematoxylin and eosin staining (H&E) in order to further determine the experimental results. In addition, we also collected 12 normal gastric tissue samples for the sake of comparison. One thing to be aware of was that in order to avoid specimen shrinkage or dehydration, we added a small amount of phosphate-buffered saline (PBS) to the tissue sections during the experiment.

### Histologic analysis

Firstly, every H&E-stained slice was checked by two certified pathologists in this study to avoid the inter-individual variability, and then a digital image of the H&E-stained slide was acquired by an optical microscope (Eclipse Ci-L, Nikon Instruments Inc., Japan) with a CCD (DS-Fi2, Nikon). Secondly, two investigators who were blinded to the diagnostic results confirmed all the multiphoton imaging results by comparing with the H&E-stained digital images.

### Statistical analysis

Furthermore, in the development of early gastric cancer, we measured the circumference of cell nucleus and SHG average intensity per pixel so that the changes of cell size and collagen content in mucosa could be quantitatively estimated. For every multiphoton image, SHG average intensity per pixel was obtained via dividing the sum of all intensities by the total number of pixels [15]. The results were presented as a mean followed with its standard deviation (mean ± SD). In this study, we made use of the ImageJ software for analyzing SHG images, and based on the IBM SPSS Statistics 21, we also employed the student’s t-test to carry out statistical analysis, and *P* value less than 0.05 was regarded as statistical significance.

## Results

### MPM imaging

**Fig. 1:**
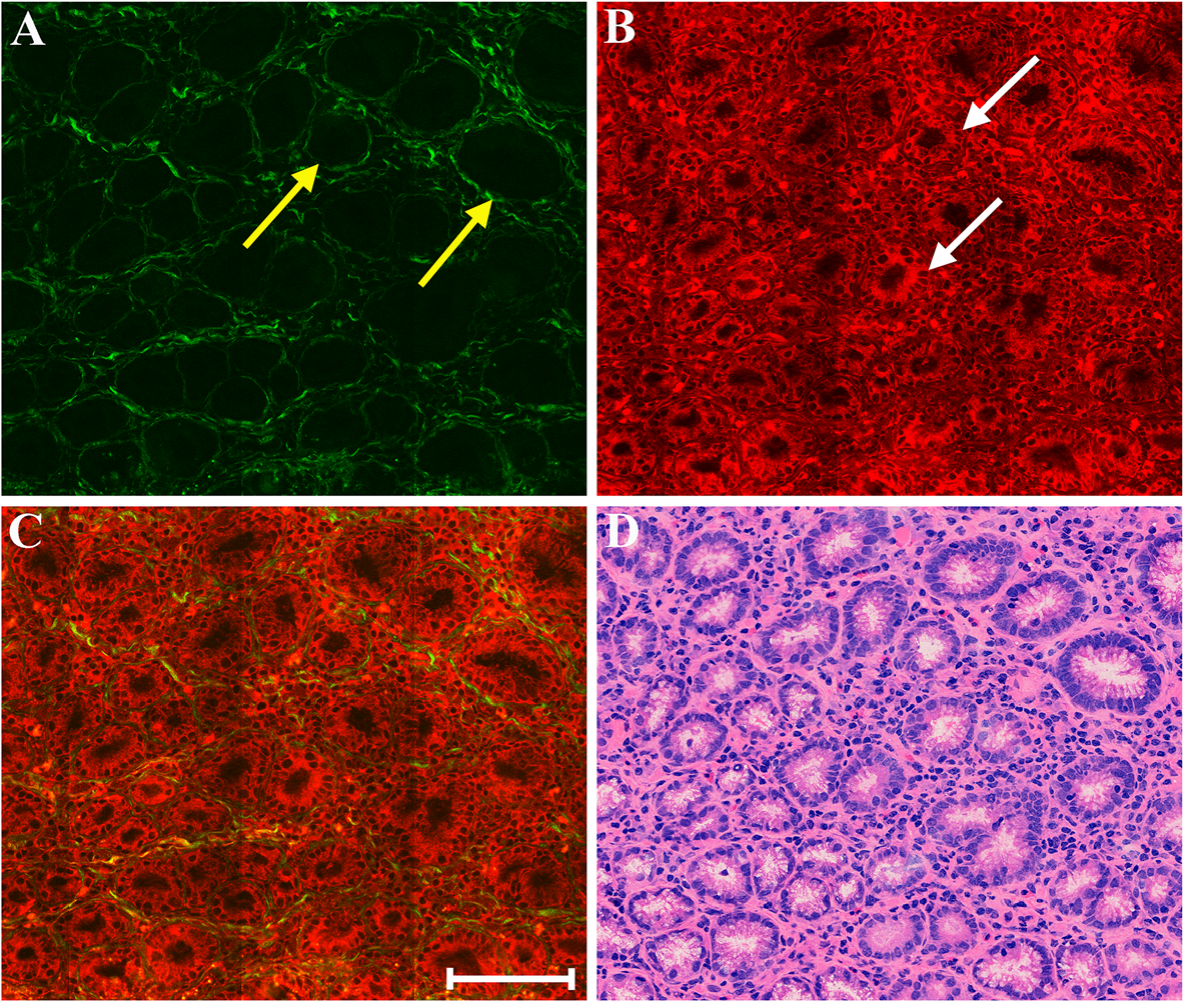
MPM images of normal gastric mucosa and corresponding H&E-stained image. **a** SHG image; **b** TPEF image; **c** Merging of SHG and TPEF images; **d** H&E-stained image. Yellow arrow: basement membrane; white arrow: epithelial cells. Scale bar: 100 μm

The focus of the first part of this work is to qualitatively determine whether MPM can effectively identify the morphologic differences between normal and early cancerous gastric tissues. Figure 1 shows representative MPM images of normal gastric mucosa and a corresponding H&E-stained image. MPM images reveal that mucosal tissues mainly consist of gastric glands and collagen fibers. Specifically, an individual epithelial cell nucleus (white arrow in Fig. 1b) which is located at basement membrane surface could be identified, and individual gastric gland composed of epithelial cells is readily differentiated via TPEF image. Normal cells are uniform in shape with small nuclei, and the gastric glands arrange regularly with uniform shape. These same details of cellular and subcellular architecture correlate well with the corresponding H&E-stained image (Fig. 1d). It is well-known that collagen matrix forms the basic framework of stroma in mucosa, and plays an important role in the development of cancer [16]. SHG image displays that the basement membrane (yellow arrow in Fig. 1a) is identified readily as a thin band surrounding individual gland, and collagen fibers in stroma form a fine reticular structure for sustaining gastric glands. Compared with the H&E-stained image, the extracellular matrix is more prominent in SHG image.

**Fig. 2:**
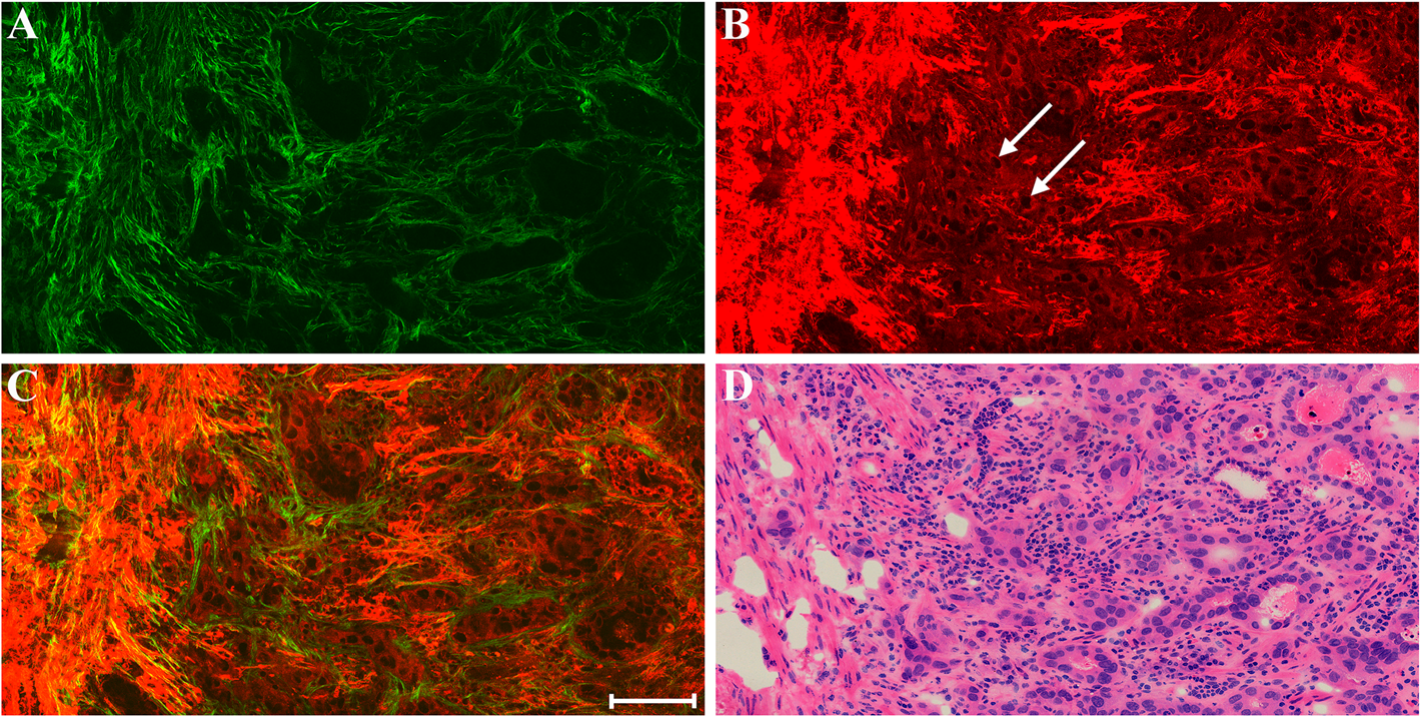
MPM images of early gastric cancer that has invaded mucosa alone and corresponding H&E-stained image. **a** SHG image; **b** TPEF image; **c** Merging of SHG and TPEF images; **d** H&E-stained image. White arrow: cancerous cells. Scale bar: 100 μm

Early gastric cancer can be divided into those that invade the mucosa only, and those that penetrate the muscularis mucosa to the submucosa. Figure 2 shows representative MPM images of early gastric cancer that has invaded mucosa alone and a corresponding H&E-stained image. TPEF image demonstrates that the morphology of individual nuclei is observed readily, and the intercellular space between individual cells could be discerned as well. Compared with normal tissues, cancerous cell nuclei grow in size significantly and cellular pleomorphism is obvious, and the gastric glands are disordered and are of various shapes.

More importantly, abnormal cells have broken through the basement membrane and invaded into the stroma sparsely and diffusely (white arrow in Fig. 2b), which makes it impossible to detect by other optical imaging techniques [17]. These features are comparable with what is obtained using standard H&E histology (Fig. 2d). SHG image displays that connective tissues which are mainly made up of collagen fibers and are used for separating individual glands could be detected too, but they are chaotic and gather together in comparison with normal gastric mucosa. The change of collagen fibers is therefore considered to reflect the development of gastric cancer.

**Fig. 3:**
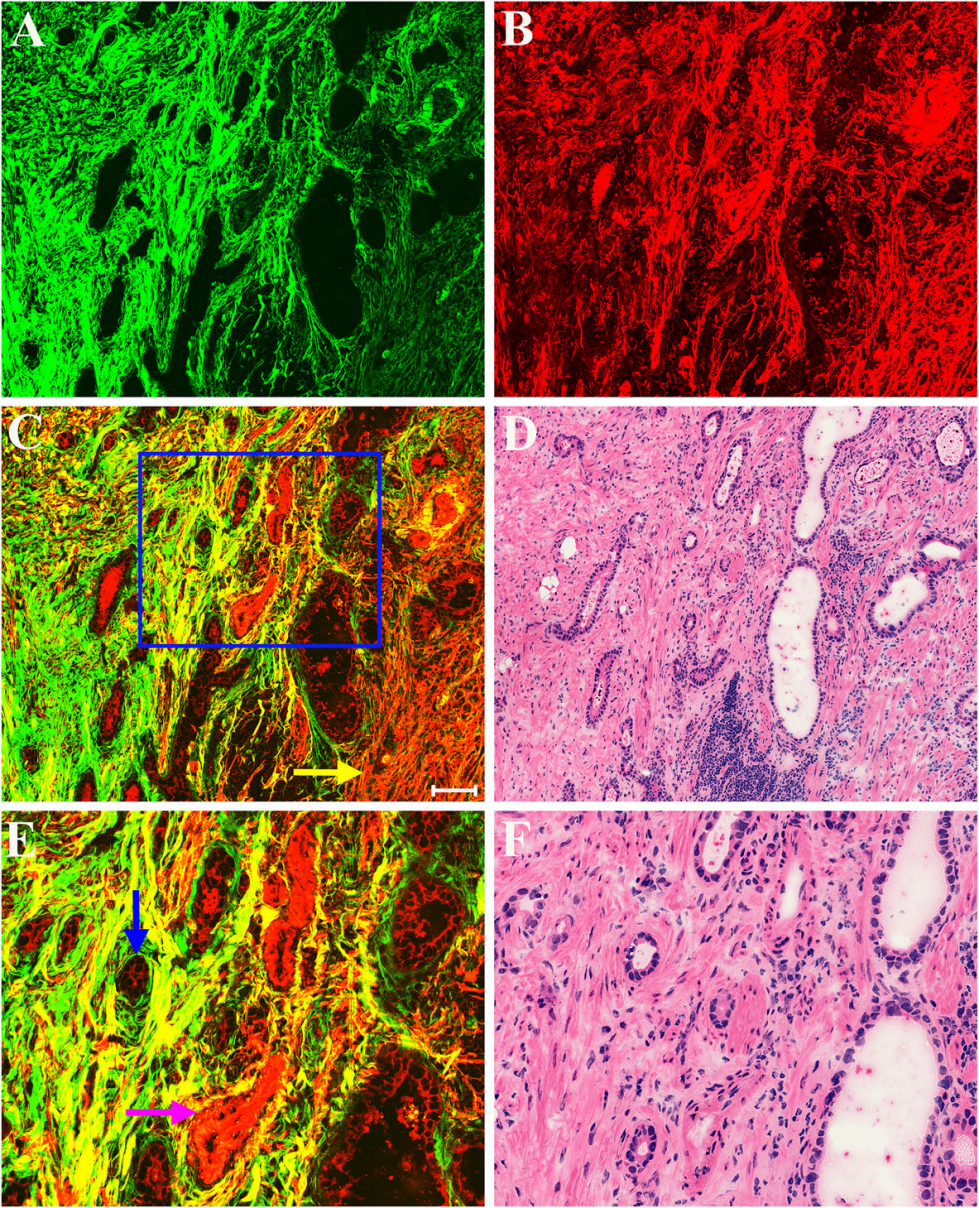
MPM images of early gastric cancer that has invaded into submucosa and corresponding H&E-stained images. **a** SHG image; **b** TPEF image; **c** Merging of SHG and TPEF images; **d** H&E-stained image; and **e**-**f** Zoom-in image of the boxed region in (**c**) and corresponding H&E-stained image. Yellow arrow: muscularis mucosae; blue arrow: tumor cells; pink arrow: blood vessel. Scale bar: 100 μm

Figure 3 shows representative MPM images of early gastric cancer that has invaded into submucosa and corresponding H&E-stained images. It can be clearly seen that malignant tumors have penetrated the muscularis mucosae (yellow arrow in Fig. 3c) to the submucosa. MPM images also reveal that the submucosal layer mainly consists of collagen fibers and elastic fibers. Thus it is easy to find tumor invasion (blue arrow in Fig. 3e) by MPM examination. Additionally, blood vessel (pink arrow in Fig. 3e) can be identified readily as elastin in the vascular wall is an endogenous fluorescent molecule, and can emit TPEF signal. These qualitative morphological variations correspond to the corresponding H&E-stained image of paired histological section.

**Fig. 4:**
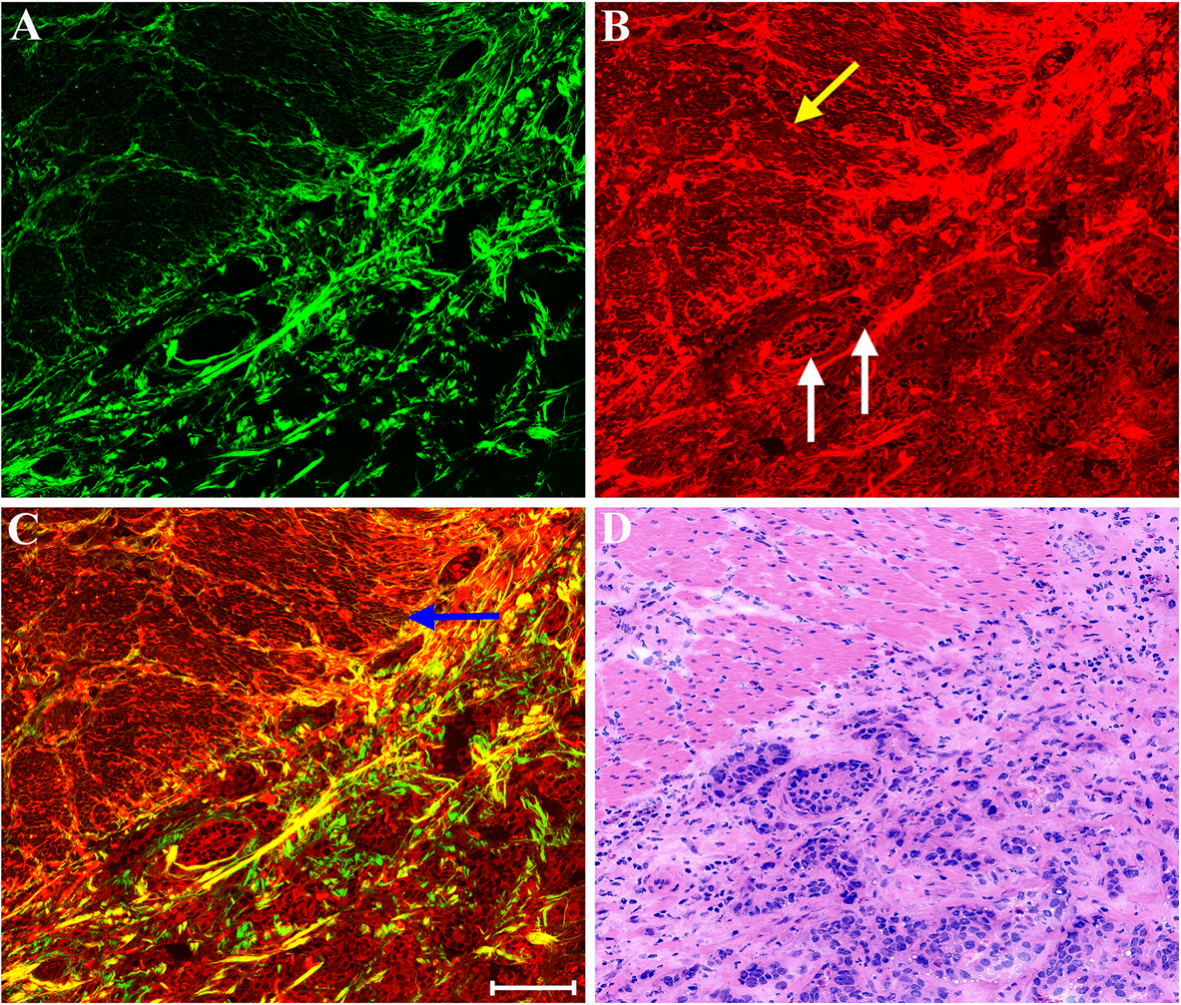
MPM images of a boundary between submucosal and muscular layers in early gastric cancer and corresponding H&E-stained image. **a** SHG image; **b** TPEF image; **c** Merging of SHG and TPEF images; **d** H&E-stained image. Yellow arrow: elastic fiber; white arrow: malignant tumors; blue arrow: muscularis propria. Scale bar: 100 μm

Figure 4 shows representative MPM images of a boundary between submucosal and muscular layers in early gastric cancer and a corresponding H&E-stained image. Morphologically, malignant tumors (white arrow in Fig. 4b) have invaded into the boundary between submucosal and muscular layers, but were still confined to the submucosa. These morphologic changes are consistent with those identified with the corresponding H&E-stained image (Fig. 4d). Unlike in normal tissues, collagen fibers in submucosa are disrupted by tumor invasion and even almost disappear. The muscularis propria (blue arrow in Fig. 4c) could be detected too by MPM image. The definition of early gastric cancer allows for invasion only as far as the submucosa, without penetration through the muscularis propria. Although tumors did not invaded into the muscular layer, elastic fibers (yellow arrow in Fig. 4b) have been affected such as fracture, aggregation. Together, these findings suggest that MPM may be useful for identifying not only normal gastric tissues but also malignant gastric lesions.

### Quantitative analysis

To determine whether these morphologic features were statistically different between normal and early gastric cancer tissues, two features in mucosa were quantified from MPM images. The nucleus circumference was measured for assessing the change in cell size, and SHG average intensity per pixel was calculated to quantify stromal collagen change. As shown in Table 1, the average and SD of normal cell nucleus circumference is 20.32 ± 2.38 μm, and of cancerous cell nucleus circumference is 47.25 ± 6.41 μm, indicating that the cell nucleus is significantly enlarged when gastric disease progresses from normal to early cancer. There is a statistically highly significant difference (*P* < 0.002) between normal and early gastric cancer.

**Table 1:** Quantitative variables for distinguishing normal and early gastric cancer tissues with multiphoton microscopy

Samples	Quantitative variables	
	Nucleus circumference (μm)	SHG average intensity per pixel
Normal (*n* = 12)	20.32 ± 2.38	16.76 ± 1.26
Early gastric cancer (*n* = 12)	47.25 ± 6.41	25.59 ± 4.47

It has become evident that collagen matrix plays an important role in the progression of early gastric cancer. Quantitative results shown in Table 1 reveal that the collagen fibers in mucosa tend to increase as gastric disease develops from normal to early cancer. More specifically, the SHG average intensity per pixel in normal mucosa is 16.76 ± 1.26, however, in cancerous mucosa is 25.59 ± 4.47. Statistically significant difference (*P* < 0.03) in the SHG average intensity per pixel was also found between normal and early gastric cancer. It is clear that the collagen content within gastric tissues is altered with the progression of early cancer, and thus the detection of the signal from collagen could provide us an approach to differentiate normal from abnormal tissues.

### Assessment of diagnostic accuracy

Lastly, we performed a blinded study to evaluate the diagnosis precision by MPM based on the optical diagnostic features. Twenty-four samples, including 12 normal cases and 12 abnormal cases were analyzed in this work. A doctor and a researcher were invited for this pilot study, and they were all blinded to the diagnosis information of the patients. The diagnosis results from the MPM and H&E staining were shown in Table 2. More specifically, the diagnosis sensitivity was 91.7%, the specificity was 100%, the positive predictive value (PPV) was 100%, and the negative predictive value (NPV) was 92.3%. The false-negative rate (FNR) was 8.3% because one early gastric cancer was diagnosed as normal case according to MPM examination. It is important to point out that the test accuracy may be limited because of limited samples and therefore further studies will need to be performed to verify these findings.

**Table 2:** Diagnosis results from the multiphoton microscopy and final histopathologic diagnosis respectively

		MPM Diagnosis		
		Normal	Cancer	
H&E Diagnosis	Normal	12	0	PPV = 100%
	Cancer	1	11	NPV = 92.3%
		Sensitivity = 91.7%	Specificity = 100%	

*Abbreviations: MPM* multiphoton microscopy, *H&E* hematoxylin-eosin, *PPV* positive predictive value, *NPV* negative predictive value

## Discussion

Most gastric cancer is diagnosed at an advanced stage with a poor survival rate, despite treatment including major surgery and adjuvant therapy. The outcome could be improved by early detection and treatment [18, 19]. Because early gastric cancer is associated with a much better prognosis than advanced disease, its diagnosis is important. Endoscopic screening is widely accepted as an available approach for early gastric cancer because it is minimally invasive, safe and convenient. However, conventional endoscopy examination has limited ability to precisely identify this lesion because of the insufficient resolution [20]. The disadvantages existing in MRT (Magnetic Resonance Imaging), CT (Computed Tomography), US (Ultrasound), and so on should not be overlooked, such as low resolution, poor contrast sensitivity, or even potential radioactive hazard [17]. Histological evaluation of biopsy specimens is still the golden standard for diagnosing early gastric cancer, but performing biopsy procedures has several shortcomings, including sampling error, costs, risks to the patient, and the delay in obtaining results [21]. All these factors together might lead to underdiagnosis or overdiagnosis and therefore suboptimal treatment as well as surveillance practices.

The advantages presented by multiphoton microscopy such as not only the low photodamage and photobleaching, high-resolution, high-sensitivity as well as high-contrast, but also providing label-free detection of biological tissues in real time, may help reduce sampling errors and even eliminate the need for invasive tissue removal [22]. TPEF from NADH as well as FAD in cells and SHG from collagen have been shown to be two major intrinsic signals for gastric mucosal tissues. MPM images show that normal gastric mucosa is mainly composed of well-aligned glands and well-organized extracellular collagen network that is essential for the maintenance of gastric glands, while abnormal mucosal tissues have disordered glands with various shapes and have chaotic extracellular collagen matrix. Stromal changes will be involved in neoplastic process for providing a tumor-promoting environment for cancer progression and metastasis [8, 23]. The presence of collagen fibers is therefore considered to influence the development of gastric cancer. As a major component of extracellular matrix, the architectural properties of collagen fibers can be obtained by SHG imaging to reflect stromal changes in abnormal tissues. Furthermore, quantitative analysis proves that the cells will be enlarged with the progression of early gastric cancer and collagen content increases because of desmoplastic response which is characterized by dense collagen growth. A pilot study showed that the diagnosis results based on MPM have high accuracy, and the sensitivity, specificity, PPV, and NPV were 91.7%, 100%, 100%, and 92.3% respectively.

Though it has been shown that collagen changes would participate in the development of cancerization [24, 25], however, interestingly enough, some researchers reported that malignant cells may secrete extracellular enzymes to degrade the collagen fibers at the invasion front [8, 26, 27], while some works demonstrated that tumor invasion may activate fibroblasts and cause desmoplasia, and therefore increase deposition of extracellular collagen [28, 29]. In this study, our data showed that collagen density increases during gastric carcinoma progression, which is consistent with the previous results [13].

The submucosa mainly consists of loose connective tissue with blood vessels, and elastic fibers as well as collagen fibers are the major components of normal connective tissue. Elastin is a common fluorescence source, and this property makes TPEF imaging a convenient tool for illustrating the microstructures of elastin fiber and blood vessel. In normal submucosal tissues, elastin fibers have a long rope-like structure, and collagen fibers are well-organized. However, in abnormal tissues, elastin fibers have almost disappeared, and collagen fibers are sparse and disrupted because malignant cells would secrete extracellular enzymes to degrade the original tissue architecture and promote tumor progression into surrounding tissues [30].

Additionally, because of the insufficient resolution of conventional endoscopy, endoscopic surgery may fail to remove disseminated invasive cells that lie beyond the surgical resection border, which may lead to tumor recurrence and ultimately patient death. The data obtained in this work show that MPM is capable of detecting single abnormal cell and identifying stratified structure in gastric tissues, and therefore may be helpful for determining surgical margin as well as selecting an optimal treatment when it is successfully incorporated into endoscope in the near future. Although the light penetration of MPM is still limited at present, it has been improving with the development of a variety of technologies, such as a gradient index lens-based MPM or a compact and flexible MPM probe [31, 32]. It was reported that the penetration depth of MPM could reach millimeter-order [33]. Maybe the combination of gradient index lens and multiphoton probe is a best method to overcome the defect of image depth.

Furthermore, the exploration of multiphoton endoscope into intravital imaging that aims to translate this technique into the clinics has been performed; especially the design of compact and flexible MPM probes for future clinical applications of gastrointestinal tract has achieved significant advancement [21, 31, 34, 35]. With miniaturization and integration of this technology [36, 37], MPM could be incorporated into endoscopy equipment or robotic surgical systems [38–40], and thus can be used in imaging during live endoscopy to diagnose gastrointestinal diseases as well as would be helpful to determine the surgical margin accurately.

## Conclusions

In summary, MPM is useful for visualizing mucosal and submucosal microstructure information with advantages over currently available endoscopy technologies, and has greater diagnostic performance for early gastric cancer. This study also shows that development of a multiphoton endomicroscope therefore has a practical meaning for performing virtual biopsies during routine endoscopic screening.
